# Natural Infection of Domestic Dogs with Raccoon Dog and Fox Amdoparvovirus During a Severe Disease Outbreak

**DOI:** 10.3390/ani16162618

**Published:** 2026-08-21

**Authors:** Vladimir Gajdov, Ivan Pusic, Sara Savic, Gospava Lazic, Marina Zekic, Vladimir Polacek, Tamas Petrovic

**Affiliations:** 1 Department of Virology, Scientific Veterinary Institute “Novi Sad”, 21000 Novi Sad, Serbia; goga@niv.ns.ac.rs; 2 Department of Epizootiology, Clinical Diagnostics, Disinfection, Disinsection and Deratization, Scientific Veterinary Institute “Novi Sad”, 21000 Novi Sad, Serbia; ivan@niv.ns.ac.rs (I.P.); vlade@niv.ns.ac.rs (V.P.); 3 Department for Immunology, Serology and Biochemistry, Scientific Veterinary Institute “Novi Sad”, 21000 Novi Sad, Serbia; sara@niv.ns.ac.rs (S.S.); marina@niv.ns.ac.rs (M.Z.)

**Keywords:** amdoparvovirus, infection, *Canis lupus familiaris*, metagenomic analysis, sequencing, cross-species transmission, novel host, wildlife, spillover, Serbia

## Abstract

Raccoon dog and fox amdoparvovirus (RFAV) has been reported in raccoon dogs and foxes, but natural infection in domestic dogs has not previously been documented. During March–April 2026, samples from four affected Dobermann dogs from a kennel near Novi Sad, Serbia, were submitted for laboratory investigation. After negative testing for canine adenovirus, canine coronavirus, herpesvirus, parvovirus, distemper virus, influenza A virus, and leptospirosis, metagenomic sequencing was performed on selected tissues, followed by bioinformatic analysis and targeted RFAV PCR screening of additional outbreak-associated samples. Affected dogs had prolonged illness characterized by conjunctivitis with ocular and nasal discharge, occasional blue eye appearance, progressive weight loss, poor coat quality, jaundice and biochemical evidence of hepatic injury, and neurologic signs including paraplegia in advanced cases. Sequencing generated 434,220 reads and identified multiple RFAV hits; pooled assembly produced a 4799 bp consensus genome with approximately 97% similarity to known RFAV strains and genome organization consistent with the genus *Amdoparvovirus.* RFAV DNA was subsequently detected by virus-specific PCR in an epidemiologically linked dog and across diverse specimen types including blood, urine, kidney, spleen, brain, lung, testicle, ileocecal lymph node, and throat swabs, whereas clinically healthy unrelated dogs were PCR-negative.

## Simple Summary

A severe disease affected four Dobermann dogs in a training facility in Serbia, near the city of Novi Sad. Symptoms in the affected dogs included eye and nasal discharge, weight loss, poor coat condition, liver abnormalities and, in some advanced cases, nervous system signs. Routine testing did not identify any of the common pathogens that could explain the disease. The aim of this study was to investigate whether an infectious agent could be associated with the observed disease. Using a method that examines all genetic material in a sample, we detected a virus previously reported in raccoon dogs and foxes. Further laboratory testing confirmed the presence of this virus in four affected dogs and in blood, urine and multiple organs, including the kidney, lung, spleen and brain. Healthy dogs from unrelated locations tested negative. These findings provide the first evidence that domestic dogs can become naturally infected with this virus. The results are important for veterinarians because they highlight a possible new infection in dogs and the need to investigate virus transmission between wildlife and domestic animals.

## 1. Introduction

Amdoparvoviruses are members of the family *Parvoviridae*, subfamily *Parvovirinae*, genus *Amdoparvovirus* [1]. They comprise a growing group of carnivore-associated viruses with small genomes of approximately 4.8 kb containing major open reading frames encoding nonstructural and capsid proteins [2]. The prototype amdoparvovirus, Aleutian mink disease virus (AMDV), is the best-characterized member and causes persistent infection with outcomes ranging from subclinical infection to fatal inflammatory disease in mink and related hosts [2]. Amdoparvoviruses are epidemiologically important because they are multi-host pathogens and several members of the genus are capable of crossing host species barriers [2,3,4]. Cross-species transmission has been documented for AMDV, skunk amdoparvovirus (SKAV), Labrador amdoparvovirus 1, and other recently described carnivore amdoparvoviruses, indicating that host plasticity is a recurring feature of this genus rather than an exception [3,4]. This broad host range is thought to be linked to macrophage tropism and antibody-dependent enhancement of infection through Fc receptor-mediated uptake of antibody-coated virions [4,5]. Amdoparvoviruses have been identified in mink, skunks, foxes, raccoon dogs, red pandas, martens, badgers, and felids, underscoring expanding host breadth [1,2,3,4,5,6,7,8]. Similar or closely related viruses have infected multiple carnivore hosts in wildlife systems, including spillover into non-maintenance hosts [6,7]. Amdoparvoviruses are linked to wasting syndromes, nephritis, vasculitis, hepatitis, pneumonia, and neurologic disease, though severity varies by host and strain [2,8,9]. Viral persistence, shedding, environmental stability, and shared habitats can create opportunities for spillover to sympatric wildlife and domestic animals [5,6]. RFAV is one of the amdoparvoviruses recognized in canids and has previously been reported in raccoon dogs and foxes [10]. The epidemiology, tissue tropism, and disease expression of newly described amdoparvoviruses outside mink remain incompletely defined [6,9]. Natural spillover of pathogens between wildlife and domestic carnivores has become a recurring concern in veterinary and conservation medicine [11,12]. Studies of carnivore viruses at human-modified or farm-linked interfaces show that domestic animals, wildlife, and captive populations can exchange pathogens, although transmission intensity varies by virus, host ecology, and contact structure [2,12,13]. Against this background, unexplained outbreaks in dogs with negative routine diagnostic testing warrant investigation for atypical or previously unrecognized agents. The present study investigated a severe outbreak of prolonged multisystemic disease in a Dobermann training facility in Serbia and aimed to investigate a potential pathogenic agent using metagenomic sequencing, characterize the detected virus genomically, and assess the distribution of viral DNA among affected dogs and specimen types.

## 2. Materials and Methods

This study was an outbreak investigation of a severe naturally occurring disease affecting epidemiologically linked Dobermann dogs kept by a professional dog trainer near Novi Sad, Serbia, during March–April 2026. The investigation combined conventional diagnostic testing, untargeted metagenomic sequencing, genome assembly and characterization, and targeted PCR screening of additional samples collected during outbreak follow-up.

### 2.1. Outbreak Investigation

The epizootiological investigation indicated that the outbreak began in December 2025, shortly after the introduction of two female Dobermanns from the Russian Federation in late November 2025. A seven-month-old female from this consignment was the first dog to develop clinical signs, within several days of arrival. Despite initial isolation of the two newly introduced dogs, disease subsequently occurred among the resident Dobermanns. At the time of the official investigation, eight dogs were present at the facility: seven Dobermanns (four females and three males; 10 months–4 years of age) and one 2.5-year-old male German Shepherd. All dogs had documented vaccination against major canine viral diseases and leptospirosis. Four of the seven Dobermanns exhibited clinical signs, while the other Dobermann dogs and the German Shepherd remained asymptomatic. The asymptomatic German Shepherd remained RFAV PCR-negative on repeated testing, as well as the other Dobermans (at the time of writing this work). Dogs were kept under confined conditions, had no known local wildlife contact, and were fed commercial dry food. Information obtained during the investigation suggested possible previous wildlife contact at the kennel of origin of the imported dogs; however, this information could not be independently verified. Therefore, the source and route of RFAV introduction into the facility could not be conclusively established.

### 2.2. Sampling and Animals

Initial submissions included samples from an adult female dog and her two puppies (kidneys, livers and lungs) which present the first suspect cases. As the outbreak progressed, additional cases were recognized and a subsequent submission from anothe affected dog included kidneys, liver, spleen, lungs, brain, intestines, tonsils, ileocecal lymph nodes, whole blood, urine, and throat and rectal swabs. Clinically affected dogs exhibited prolonged disease with ocular and nasal discharge, conjunctivitis, occasional blue eye appearance, progressive weight loss, poor coat quality, jaundice, biochemical evidence of hepatic injury, and neurologic signs in advanced stages.

### 2.3. Diagnostic Testing

Because the clinical syndrome was nonspecific, testing for several common and diagnostically relevant canine infectious diseases was performed. Samples were tested by PCR/qPCR on repeated occasions for canine adenovirus, canine coronavirus, herpesvirus, parvovirus, distemper virus, influenza A virus, and leptospirosis.

### 2.4. Nucleic Acid Extraction and Metagenomic Sequencing

After routine tests were unrevealing, metagenomic high-throughput sequencing was used to search for a candidate etiologic agent. Nucleic acids (RNA and DNA) were extracted using the IndiSpin Pathogen Kit (Qiagen, Hilden, Germany) from lung, kidney, and liver tissues from an affected dam and pups and from kidneys, liver, spleen, lungs, brain, intestines, tonsils, ileocecal lymph nodes, whole blood, urine, and throat and rectal swabs from the other affected dog. Samples from the dam and pups (liver, kidney and lungs) underwent first-strand cDNA synthesis using RevertAid First-Strand cDNA Synthesis Kit (Thermo Fisher Scientific, Waltham, MA, USA) with random hexamer primers. The obtained cDNA was quantified using a Qubit 4.0 instrument, and libraries were prepared using the Rapid Barcoding Kit V14 (Oxford Nanopore, Oxford, UK, SQK-RBK114.24) according to the manufacturers’ instructions. Sequencing was conducted on an Oxford Nanopore MinION Mk1D device.

### 2.5. Bioinformatics and Phylogenetics

A total of 434,220 sequencing reads were generated. Reads were filtered using NanoFilt v. 2.8.0 [14] with quality threshold ≥ 9 and minimum length ≥ 200 bp. Host reads were removed by mapping against the canine reference genome (UU_Cfam_GSD_1.0, RefSeq assembly GCF_011100685.1) using minimap2 v. 2.30 [15] and SAMtools v. 1.21 [16]. Cleaned reads were searched against a custom-made viral-only DIAMOND v. 2.1.13. [17] database (containing all viral sequences available in NCBI’s GenBank, accessed on 28 April 2026), which yielded multiple amdoparvovirus hits in each sample. Individual *de novo* per sample assemblies with Flye v. 2.9.6 [18] generated fragmented genomes consisting of two major contigs representing the terminal genomic regions, missing the central portion of the genome. Pooling Nanopore reads from all three samples increased effective sequence assembly, followed by two polishing rounds with Medaka v. 1.2.9 [19] to produce a final near-complete 4799 bp consensus genome, deposited in GenBank under accession number PZ348355. BLASTn (https://blast.ncbi.nlm.nih.gov/Blast.cgi?PROGRAM=blastn&PAGE_TYPE=BlastSearch&LINK_LOC=blasthome, accessed on 1 July 2026) analysis showed approximately 97% similarity to known RFAV sequences, and genome organization was consistent with amdoparvoviruses, including two major ORFs encoding NS1 and VP proteins and an additional smaller ORF. Phylogenetic analysis was performed using whole-genome nucleotide sequences. Reference sequences representing available RFAV strains were retrieved from GenBank. Sequences were aligned using MAFFT v. 7.5 [20]. Maximum-likelihood phylogenetic analysis was performed using IQ-TREE v. 2.4.0 [21]. The best-fitting nucleotide substitution model was selected using ModelFinder. Branch support was assessed using 1000 ultrafast bootstrap replicates and 1000 SH-aLRT replicates, with BNNI optimization. The tree was rooted using Aleutian mink disease virus as the outgroup and visualized using iTOL v. 6.0 [22]. After quality filtering, 144,076, 46,629, and 148,869 reads remained for Samples 1–3, respectively. Host depletion against the canine genome retained 13,019, 1734, and 14,866 non-host reads. Mapping of the cleaned reads to the pooled RFAV consensus genome identified 18, 148, and 56 primary RFAV-associated reads, corresponding to genome coverages of 73.4%, 100%, and 100%, with mean depths of 3.75×, 24.22×, and 10.39× for Samples 1–3 (lung, liver, and kidney), respectively.

### 2.6. PCR Screening

Following metagenomic identification of RFAV, an amdoparvovirus-specific PCR as described by Shao et al. [10], using primer pair AV7, including the published primer sequences, target region and amplification conditions, was applied to additional samples collected during the outbreak investigation. PCR used the HotStarTaq Master Mix Kit (Qiagen, Hilden, Germany) with annealing at 53 °C for 30 s. Each 25 µL reaction contained 12.5 µL HotStarTaq Master Mix, 0.8 µM of each primer, and 5 µL DNA template.

## 3. Results

**Figure 1:**
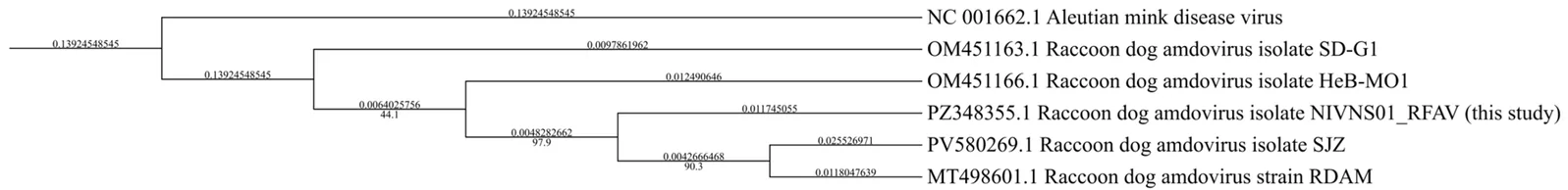
Maximum-likelihood phylogenetic tree based on whole-genome nucleotide sequences of RFAV. Sequences were aligned using MAFFT v. 7.5, and the tree was inferred using IQ-TREE v. 2.4.0. Branch support was assessed using 1000 SH-aLRT replicates and 1000 ultrafast bootstrap replicates; support values are shown at the nodes as SH-aLRT/UFBoot (%). The tree was rooted using Aleutian mink disease virus as the outgroup.

Clinical illness in affected Dobermann dogs was prolonged and multisystemic rather than peracute. The clinical course varied between dogs, with an estimated incubation period ranging from approximately two weeks to two months. Common findings included bilateral mucopurulent conjunctivitis and rhinitis, marked conjunctival and scleral injection, and, in later stages, corneal edema with diffuse opacity (“blue eye”). Progressive weight loss despite preserved appetite, poor coat quality, jaundice, and biochemical evidence of hepatic injury, predominantly increased transaminase activities, were also observed. Additional findings included hyperkeratosis of the footpads and nasal planum, coughing, and arthritis. In advanced cases, neurological signs included epileptiform seizures, ataxia, and hemiparesis. Terminal cachexia and organ failure were frequent reasons for death or euthanasia. Gross postmortem lesions involved multiple parenchymatous organs and were most pronounced in the liver, kidneys, and pancreas. Repeated PCR targeted testing did not identify common canine viral or bacterial causes considered relevant to the presentation. Samples were negative for canine adenovirus, canine coronavirus, herpesvirus, parvovirus, distemper virus, influenza A virus, and leptospirosis on several testing occasions. Metagenomic sequencing was pursued after routine diagnostics were repeatedly negative. De novo assembly from lung, liver and kidney samples yielded contigs with approximately 97% BLASTn similarity to raccoon dog and fox amdoparvovirus. Pooling of cleaned reads enabled reconstruction of a polished 4799 bp consensus genome, and genome annotation showed the expected amdoparvovirus organization with major ORFs encoding NS1 and VP proteins plus a smaller ORF. Phylogenetic analysis supported classification of the strain as a separate RFAV variant (Figure 1). Targeted PCR extended the metagenomic finding to additional outbreak-associated animals and specimen types. RFAV DNA was detected in whole blood, urine, kidney, spleen, brain, lung, testicle, ileocecal lymph node, and throat swabs from an epidemiologically linked Dobermann dog. In contrast, samples from five clinically healthy dogs from unrelated locations and different breeds were PCR-negative, and no amplification was seen in tested samples (internal organs taken from our sample bank) from birds, pigs, bovines and cats used for confirmation of specificity.

## 4. Discussion

This study provides evidence that domestic dogs can be naturally infected with RFAV, originally described in farmed raccoon dogs and arctic foxes, while subsequent studies have identified RFAV or closely related viruses in additional carnivore hosts [6,10]. Detection in domestic dogs therefore further expands the recognized host range of this virus. Infection of dogs is biologically plausible given the multi-host ecology of amdoparvoviruses. Closely related viruses have been detected in different carnivore species, including maintenance and spillover hosts [3,5,6]. However, the mechanism enabling RFAV infection of dogs was not investigated in the present study. The clinical presentation of the affected dogs showed some similarities to diseases associated with other amdoparvoviruses. Wasting and renal involvement have been described in Aleutian disease in mink and ferrets, while renal, vascular and neurological lesions have been associated with SKAV infection in striped skunks [8,13,23]. Meningoencephalitis and viral detection in the brain have been reported in AMDV-infected mink and SKAV-infected skunks [8,23]. These observations provide a relevant comparison with the neurological manifestations observed in the present outbreak and the detection of RFAV DNA in brain tissue. Detection of RFAV DNA in blood, urine and multiple internal tissues, including lymphoid tissue, kidney, lung and brain, supports systemic viral distribution. Detection in throat swabs and urine is epidemiologically relevant because SKAV has been localized to gastrointestinal, urinary-tract and skin epithelium, suggesting several potential routes of shedding [7]. Several limitations should therefore be considered. The investigation included a limited number of outbreak-associated animals, and complete clinical and pathological data were not available for all dogs. The study did not include histopathological colocalization of RFAV with lesions, viral isolation, serological testing or longitudinal measurement of viral loads. The findings therefore establish natural RFAV infection in domestic dogs and identify a plausible association with multisystemic disease.

## 5. Conclusions

We identified an RFAV variant in multiple Dobermann dogs during a severe disease outbreak in Serbia, providing evidence that domestic dogs can become naturally infected with RFAV. The detection of viral DNA in multiple tissues and epidemiologically linked animals suggests that RFAV is potentially associated with the onset of clinical signs in dogs and supports the possibility that RFAV has crossed the host species barrier into domestic dogs. Additional studies are underway to determine the pathogenicity, epidemiology, and geographic distribution of RFAV in domestic and wild canids in Serbia.

## Data Availability

The sequence data presented in the study are openly available NCBI’s GenBank under the accession number: PZ348355.
